# Patients infected with *Mycobacterium africanum* versus *Mycobacterium tuberculosis* possess distinct intestinal microbiota

**DOI:** 10.1371/journal.pntd.0008230

**Published:** 2020-05-13

**Authors:** Sivaranjani Namasivayam, Bassirou Diarra, Seydou Diabate, Yeya dit Sadio Sarro, Amadou Kone, Bourahima Kone, Mohamed Tolofoudie, Bocar Baya, Mahamane T. Diakite, Ousmane Kodio, Keira Cohen, Jane Holl, Chad J. Achenbach, Soumya Chatterjee, Robert Leo Murphy, William Bishai, Souleymane Diallo, Alan Sher, Mamoudou Maiga

**Affiliations:** 1 Immunobiology Section, Laboratory of Parasitic Diseases, National Institute of Allergy and Infectious Diseases, National Institutes of Health, Bethesda, Maryland, United States of America; 2 University of Sciences, Techniques and Technologies of Bamako (USTTB), Bamako, Mali; 3 Johns Hopkins University School of Medicine, Baltimore, Maryland, United States of America; 4 Northwestern University, Institute for Global Health, Chicago, Illinois, United States of America; 5 Saint Louis University, Division of Infectious Diseases, Allergy & Immunology, Saint Louis, Missouri, United States of America; QIMR Berghofer Medical Research Institute, AUSTRALIA

## Abstract

**Background:**

*Mycobacterium tuberculosis* complex (MTBC), the causative agent of tuberculosis (TB), is composed of eight subspecies. TB in West Africa, in contrast to other geographical regions, is caused by *Mycobacterium africanum* (MAF) in addition to *M*. *tuberculosis* (MTB), with both infections presenting similar symptoms. Nevertheless, MAF is considered to be hypovirulent in comparison with MTB and less likely to progress to active disease. In this study, we asked whether MAF and MTB infected patients possess distinct intestinal microbiomes and characterized how these microbiota communities are affected by anti-tuberculosis therapy (ATT). Additionally, we assessed if the changes in microbiota composition following infection correlate with pathogen induced alterations in host blood-gene expression.

**Methods:**

A longitudinal, clinical study of MAF infected, MTB infected patients assessed at diagnosis and two months after start of ATT, and healthy, endemic controls was conducted to compare compositions of the fecal microbiome as determined by 16S rRNA sequencing. A blood transcriptome analysis was also performed on a subset of subjects in each group by microarray and the results cross-compared with the same individual’s microbiota composition.

**Findings:**

MAF participants have distinct microbiomes compared with MTB patients, displaying decreased diversity and increases in Enterobacteriaceae with respect to healthy participants not observed in the latter patient group. Interestingly, this observed elevation in Enterobacteriaceae positively correlated with enhanced inflammatory gene expression in peripheral blood and was reversed after initiation of ATT.

**Interpretation:**

Our findings indicate that MAF and MTB have distinct associations with the gut microbiome that may be reflective of the differential susceptibility of West Africans to these two co-endemic infections either as biomarkers or as a contributing determinant.

## Author summary

*Mycobacterium africanum* (MAF) is a hypovirulent mycobacterium species that is co-endemic with *Mycobacterium tuberculosis* (MTB) in West Africa and is selectively responsible for up to half the tuberculosis cases in this region. Why some individuals become infected with MAF versus MTB is unclear but has been suggested to be determined by differential host immune competency. Since the microbiome has now been implicated in numerous studies to generally influence host resistance to disease, we investigated whether differences in the intestinal microbiota might associate with MAF as compared with MTB infection. This report presents the first analysis of the intestinal microbiome of MAF-infected subjects as well as a comparison with the microbiota of co-endemic MTB patients and reveals that the microbiota of individuals with MAF infection display both decreased diversity and distinct differences in microbial taxa when compared to both MTB-infected and healthy controls. Furthermore, our data reveal for the first time in TB patients a correlation between the abundance of certain taxa and host blood transcriptional changes related to immune function. Our study also establishes that antibiotic treatment induces parallel changes in the gut microbiota of MAF- and MTB-infected patients. Although not directly addressed in the present study, the findings presented here raise the possibility that the microbiota or other host physiologic or immune factors closely associated with it may be a factor underlying the differential susceptibility of West Africans to MAF infection. In addition, the data identify certain commensal taxa that could be tested in future studies as specific determinants of this association.

## Introduction

Tuberculosis (TB) continues to result in substantial morbidity and mortality worldwide, with 1.6 million deaths in 2017 alone, more than any other single infectious disease [1]. Most of these deaths occur in developing countries in Africa and Asia. In West Africa, *Mycobacterium africanum* (MAF) accounts for up to half of TB cases, but is rarely involved in TB infections in other regions [2–5]. MAF belongs to the *Mycobacterium tuberculosis* complex (MTBC), which contains six other lineages including *M*. *tuberculosis sensu stricto* (MTB). First isolated in Senegal in 1968, MAF is considered to be less virulent than MTB and its infections are less-likely to progress to active disease or to develop drug resistance in comparison with MTB [6, 7]. Also, MAF is more likely to cause TB in older, malnourished and HIV-coinfected individuals [8, 9]. This predilection of MAF for West Africans and “compromised subjects,” such as immunodeficient individuals, is poorly understood and affords a unique, natural scenario in which to investigate differential human susceptibility to TB.

Recently, the microbiome has emerged as an important host-associated factor influencing the outcome of infectious and inflammatory diseases [10–13]. Studies in murine models, utilizing broad-spectrum antibiotics or *Helicobacter hepaticus* to induce a dysbiosis, have demonstrated a potential role for the intestinal microbiome in host susceptibility to MTB [14–16]. Yet, minimal alterations in the composition of the microbiome have been observed following murine MTB infection [17, 18]. Similarly, studies in humans have identified differences in microbial taxa in TB patients in comparison to healthy controls [19–25]. Interestingly, *Helicobacter pylori* in patients, in contrast to *H*. *hepaticus* in mice, has been associated with protection against TB [26]. Nevertheless, the role of the microbiome in human TB disease and severity still remains poorly understood [21, 22]. One approach to address this question would be to characterize the microbiota in patient populations exhibiting different disease manifestations. The study of TB disease in West Africa, where hypovirulent MAF is co-endemic with the more virulent MTB, offers an opportunity to perform such an analysis.

Although, ten million patients receive TB drugs annually worldwide, the impact and consequences of antituberculosis drugs on the gut microbiota has only recently been investigated. Using a mouse model, we showed that first-line TB therapy, consisting of two months of isoniazid (H), rifampin (R), pyrazinamide (Z) and four months of HR (2HRZ/4RH), profoundly alters the gut microbiome, generating a long lasting dysbiosis after treatment cessation [18]. Similar alterations have been reported in cross-sectional studies of TB patients, with the resulting dysbiosis lasting for more than a year after the conclusion of therapy [27, 28]. However, the effects of antituberculosis therapy (ATT) on the microbiome of MAF infected patients have not been characterized nor have the changes been compared with those occuring longitudinally in treated MTB infected individuals.

In this study, we characterize and compare the intestinal microbiomes of patients with MAF or MTB infections before and during ATT in Mali, West Africa. We found that MAF infected patients displayed decreased microbiota diversity and elevated levels of certain taxa, when compared with both MTB infected patients and healthy controls. Nevertheless, ATT resulted in similar alterations in microbiota composition in both MAF and MTB infected groups. Blood transcriptome profiling has been previously employeed to identify signatures of active TB disease as well as markers of latent to active TB disease progression that have provided valuable mechansitic insights into understanding the host-resistance pathways involved [29–31]. Therefore, we sought to explore possible associations between host blood gene expression and the microbiota communities in this West African cohort. Interestingly, correlation analyses performed on data from the MAF, MTB, and control groups revealed that Enterobacteriaceae levels were positively correlated with a subset of immune-response related genes elevated during infection while several other taxa were negatively correlated with the same peripheral blood transcripts. Taken together, the findings of this study reveal an association of the different diseases induced by MAF versus MTB with the composition of the intestinal microbiome and provide preliminary evidence for a link between the host immune response and the structure of the gut flora in these patients.

## Methods

### Ethical approvals

The study protocol was approved by the ethics committee of the Faculty of Medicine and Odontostomatology of the University of Sciences, Technics and Technologies of Bamako (USTTB), Mali (approval number: 2014/04 CE/FMPOS) and the Institutional Review Board (IRB) of Northwestern University (approval number: STU00094500). Written informed consent was obtained from all study participants before enrollment.

### Subjects

Adult individuals (≥18 years of age) with active pulmonary TB (defined by positive sputum culture) and healthy volunteers were prospectively recruited on a rolling basis over a two-year time period at the Point-G University Teaching Hospital of Bamako, in Mali. To meet study inclusion criteria, healthy controls had to be asymptomatic and to have a negative QuantiFERON-TB Gold test result to confirm no active or latent TB infection. The TB groups were composed of newly infected patients who were eligible for first-line TB ATT, (2HRZE/4RH). A total of 20 MAF and 21 MTB infected patients and 10 healthy controls were included in the microbiome analysis. The TB-infected patients were recruited based on enrollment sequence of the first 20 MAF and first 21 MTB eligible and consenting patients from a cohort of 100 patients. Blood transcriptome analysis was performed on the 10 healthy participants and the first 10 patients enrolled in each of the infected (MAF or MTB) groups.

### Sample size calculation

Sample size calculations were conducted using the Fleiss Method. We estimated, *a priori*, that 20 participants each would be needed in the MAF and MTB groups to achieve 80% power with a two-sided significant level (α) of 0.05, and at a 95% confidence level. We assumed based on previous studies [4, 32], that 42% of patients are exposed/likely to be MAF-infected, with an Odds Ratio of 14 (confidence interval: 11–17), a risk/prevalence ratio of 8.5 and a risk/prevalence difference of 37.

### Study design and procedures

The TB patients had two study visits, one before treatment and one after two months of ATT. Healthy controls had only one study visit. All patients and controls completed a standardized interview to obtain demographic information, symptoms and details regarding prior TB and/or HIV treatment and current medications. Patients and controls all underwent a physical examination. Early morning sputum and stool samples were obtained from patients and all patients and controls were tested for HIV by Determine HIV-1/2 (Abbott Laboratories, Matsudo-Shi, Chiba, Japan). Stool samples were stored in a -80 degrees Celsius freezer within two hours of collection and the sputum samples were cultured and speciated at the NIH-funded and certified-Biosafety Laboratory Level-3 (BSL-3) at the SEREFO HIV/TB Laboratories of USTTB.

### Mycobacterial culture and identification

Sputum specimens were digested and decontaminated using the standard N-Acetyl-L-Cysteine/4% NaOH method, before inoculating both liquid (*Mycobacterium* Growth Incubator Tube [BBL^™^ MGIT^™^ Becton Dickinson, Sparks MD, United States of America), and solid (Middlebrook 7H11 Agar and Selective 7H11 Agar) media. Simultaneously, an aliquot of concentrated specimen was prepared for indirect commercial Auramine/Rhodamine staining (BBL^™^ Becton Dickinson, Sparks MD, USA). Positive cultures were confirmed to belong to the MTBC by acid-fast bacilli and nucleic acid probe (AccuProbe GenProbe, San Diego, CA, USA). Spoligotyping was performed using a commercial kit (Isogen Life Science, Netherlands) in order to identify MAF and MTB species as previously described [33] and the patients were retrospectively classified into either MAF or MTB groups. No dual MAF and MTB infections were detected in patients.

### Stool DNA extraction, microbiome sequencing and analysis

DNA was extracted from the stored stool samples using QIAamp Fast DNA Stool Mini kit (Qiagen, Hilden, Germany) according to the manufacturer’s recommendations and shipped to the NIH for sequencing. Extracted DNA was quantified using a spectrophotometer (Thermo Scientific NanoDrop 1000). The V4 region of the 16S rRNA gene was amplified using the primers (5'-TCGTCGGCAGCGTCAGATGTGTATAAGAGACAGGTGCCAGCMGCCGCGGTAA-3’ and 5'-GTCTCGTGGGCTCGGAGATGTGTATAAGAGACAGGGACTACHVGGGTWTCTAAT-3’) and sequenced using the Illumina MiSeq as previously described [18, 34]. The sequenced demultiplexed paired-end fastq reads were processed and analyzed using QIIME2 version 2–2018.4 [35]. The DADA2 algorithm, implemented in QIIME2 was used for error modelling and filtering the raw fastq files [36]. After denoising and chimera removal we obtained a total of 3,430,875 reads from 92 samples (51 for the three groups at study visit 1 and 41 for the two infection groups at study visit 2 at 2 months following start of ATT) with an average of 37,292 reads per sample. One sample each from the MAF and MTB infected groups yielded very few reads and were not included in further analyses. Thus, a final number of 10 healthy participants, 19 MAF and 20 MTB patients were included in the microbiome analyses.

Taxonomic classification was performed using Silva database release 132 [37]. The samples were rarefied at a sampling depth of 10,000 reads for alpha and beta-diversity analyses. Alpha-diversity was estimated using the Shannon index and statistical significance was assessed using a non-parametric t-test. Bray-Curtis dissimilarity index was utilized to estimate beta-diversity and visualized on a two-dimensional principle component plot generated using ggplot in R [38]. PERMANOVA with 999 permutations was used to test the statistical significance of differences in the clustering pattern between two groups. To estimate significant differences between taxa, the microbiome composition data were tested for normal distribution and the parametric Student’s t-test were utilized for normally distributed data and the non-parametric Mann Whitney U test used if the data do not follow a normal distribution pattern. Linear discriminant analysis (LEfSe), a statistical tool that performs high dimensional class comparisons on metagenomic data and provides an estimation (linear discriminant analysis, LDA, score) of the magnitude of the observed differences, was used to identify differentially abundant taxa in pairwise comparisons of the three groups [39]. Taxa with a LDA score of > 2 and p-value of < 0.05 were considered statistically different. The Wilcoxon paired non-parametric test was utilized in the longitudinal comparisons of microbiota composition before diagnosis and two months after ATT. Statistical tests and significance values associated with each analysis are indicated in the figures and figure legends.

### Blood RNA extraction and microarray analysis

RNA was isolated from peripheral blood collected in PAXgene blood RNA tubes (PreAnalytiX, Hombrechtikon, Switzerland) using a QIAamp RNA Blood extraction Kit (QIAGEN Inc., Germantown, USA). Microarray hybridization was performed using human Affymetrix Clariom D (Applied Biosystem Inc., Foster Coty, USA). Microarray gene expression data was analyzed using the Partek Genomics Suite 7.0.1 (Partek Ink. St. Louis, MO, USA). Raw data were normalized using the robust multi-array average (RMA) approach. Two-way ANOVA was utilized to identify differentially expressed genes between two groups and genes with a fold change of greater than 2 and p-value < 0.01 following Benjamini-Hochberg correction for multiple testing were considered statistically significant and utilized for further analyses. Gene Ontology (GO) term enrichment analysis was performed using Fisher’s exact test on these differentially expressed genes to identifying biological processes that were enriched. Similarly, Ingenuity Pathway Analysis (Qiagen Inc., https://www.qiagenbioinformatics.com/products/ingenuitypathway-analysis) was performed to identify pathways upregulated or downregulated in the two disease groups in comparison to the healthy group. Genes classified under immunological, inflammatory, infectious and metabolic diseases as well as immune cell trafficking, antimicrobial response, humoral immune response, cell-mediated response and antigen presentation were identified, and utilized for hierarchical clustering. Unsupervised hierarchical clustering of the normalized expression values from each participant was performed using Partek and only 50 genes with the most fold change in either direction (upregulated or downregulated) are represented as a heatmap for better visualization.

### Correlation analyses of the intestinal microbiota composition and blood gene expression

Paired correlation analysis, using the Spearman correlation method, was performed between the relative abundance of family-level taxa and the normalized expression value of annotated immune-pathway related genes that were altered during infection in comparison to healthy controls. Each pairwise correlation for the MAF group was calculated using a pool of 9 healthy controls and 8 MAF patients for whom both microbiome and transcriptome data were available. Similarly, correlations for the MTB group were calculated using the same 9 healthy controls and 9 MTB-infected patients. Significance estimates from the correlation analysis were corrected for multiple testing using the Benjamini-Hochberg procedure. The correlation matrix was clustered using unsupervised hierarchical clustering and displayed as a heatmap. Correlation analyses and visualization were performed using R.

## Results

### Demographic and clinical characteristics of study participants

This study included 10 healthy controls, 21 MTB- and 20 MAF-infected patients. Demographic and clinical characteristics for each of the three groups are described in Table 1. Overall, the groups were comparable in gender distribution and age range. In addition, the two TB groups were similar in terms of smoking status and inner-city or suburban residence. Disease severity determined by chest x-ray, sputum smear and culture results were also highly similar with no significant differences between the two TB-infected groups. All participants are Malian residents. HIV seropositive individuals were excluded from this study because of the impact of HIV infection on the immune response, which would bias our analysis. Similarly, patients with multi-drug resistant TB (MDRTB) were also excluded. Based on these criteria, 4 HIV positive and 3 MDRTB patients were excluded.

**Table 1:** Sociodemographic analysis of study participants.

Parameter		*M*. *tuberculosis* N = 21 n (%)	*M*. *africanum* N = 20 n (%)	Controls N = 10 n (%)	p-value^$^
**Gender—Male**		19 (90.48)	14 (70)	6 (60)	0.312
**Age**	[18–30]	11 (52.38)	9 (45)	8 (80)	0.435
	[30–45]	10 (47.62)	8 (40)	1 (10)	-*
	[45–60]	0 (0)	2 (10)	1 (10)	-
	[60–75]	0 (0)	1 (5)	0 (0)	-
**Smoking (current and past)**	Yes	10 (47.62)	4 (20)	n/a	0.797
**Inner-city or sub-urban**	Yes	18 (85.71)	17 (85)	9 (90)	0.710
**House hold contacts**	Yes	3 (14.29)	7 (35)	n/a	-
**Sputum smear at M0**^#^	Many AFB	20 (95.24)	16 (80)		0.440
**Sputum smear at M2**^#^	Moderate AFB	8 (38.10)	7 (35)		0.854
**Sputum culture at M0**^#^	Positive	21 (100)	20 (100)	n/a	-
**Sputum culture at M2**^#^	Positive	3 (14.29)	1 (5)		-
**Chest X-rays**	Bilateral infiltrate	3 (14.28)	7 (35)	n/a	-
	Cavitary lesions	4 (14.28)	1 (5)	n/a	-
	Miliary pattern	1 (4.76)	0 (0)	n/a	-
	Unilateral infiltrate	6 (28.5)	5 (25)	n/a	0.852

^$^p-value was calculated using Chi-square test

*p-value not calculated when n was less than 5

^#^M0- At the time of diagnosis before start of ATT; M2- Two months after start of ATT

### Association of MAF infection with decreased diversity and increased abundance of Enterobacteriaceae

Previous studies in mice and humans have reported minor alterations in the intestinal microbiota associated with MTB infection [17–20]. Whether any changes occur in the microbiome composition during MAF infection was unknown. To address this question, we compared the intestinal microbiota of MAF-infected with MTB-infected patients as well as with healthy controls. Fecal samples were collected from the patients at the time of initial diagnosis before start of ATT and the V4 region of the 16S rRNA gene was sequenced to analyze the composition of the microbiota.

**Fig 1:**
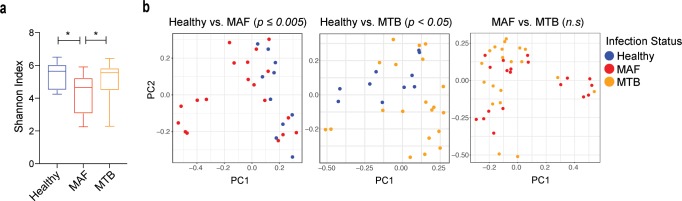
Effect on microbiome diversity and community structure following TB disease caused by MAF and MTB. a. Alpha diversity in the healthy, MAF and MTB groups were estimated using the Shannon index. Error bars indicate minimum and maximum values. Significant differences are indicated. *p < 0.05 (Mann-Whitney U test). b. Beta-diversity estimates were calculated using the Bray-Curtis dissimilarity index and represented here on a principal component (PC) plot. Each circle denotes a single patient and is color-coded by group as indicated in the key. Statistical significance was calculated using PERMANOVA with 999 permutations and is indicated for each comparison (*n*.*s*.–not significant).

We first measured the microbiota diversity within each patient using the Shannon alpha diversity index (a measure of the richness and distribution of the microbiota taxa within a sample) and found that the diversity of the microbiota in MAF-infected patients was significantly lower in comparison to MTB- infected patients and healthy controls (Fig 1A). In contrast, MTB infection was not significantly associated with a decrease in alpha diversity (Fig 1A). Next, we performed pair-wise beta diversity clustering analyses (a measure of microbiota diversity between groups) using Bray-Curtis dissimilarity matrix to compare the bacterial community structure between the three groups. The microbiota data from MAF-infected patients clustered separately from that of healthy controls (Fig 1B). Similarly, MTB-infected patients had a significant difference in microbiota community structure in comparison to healthy controls. Nevertheless, the beta-diversity of the bacterial communities of the MAF- and MTB-infected patient groups were not significantly different from each other. Further analysis failed to reveal any association of the demographic and clinical parameters such as gender, smoking status, and weight with the microbiome community structure (S1 Fig).

**Fig 2:**
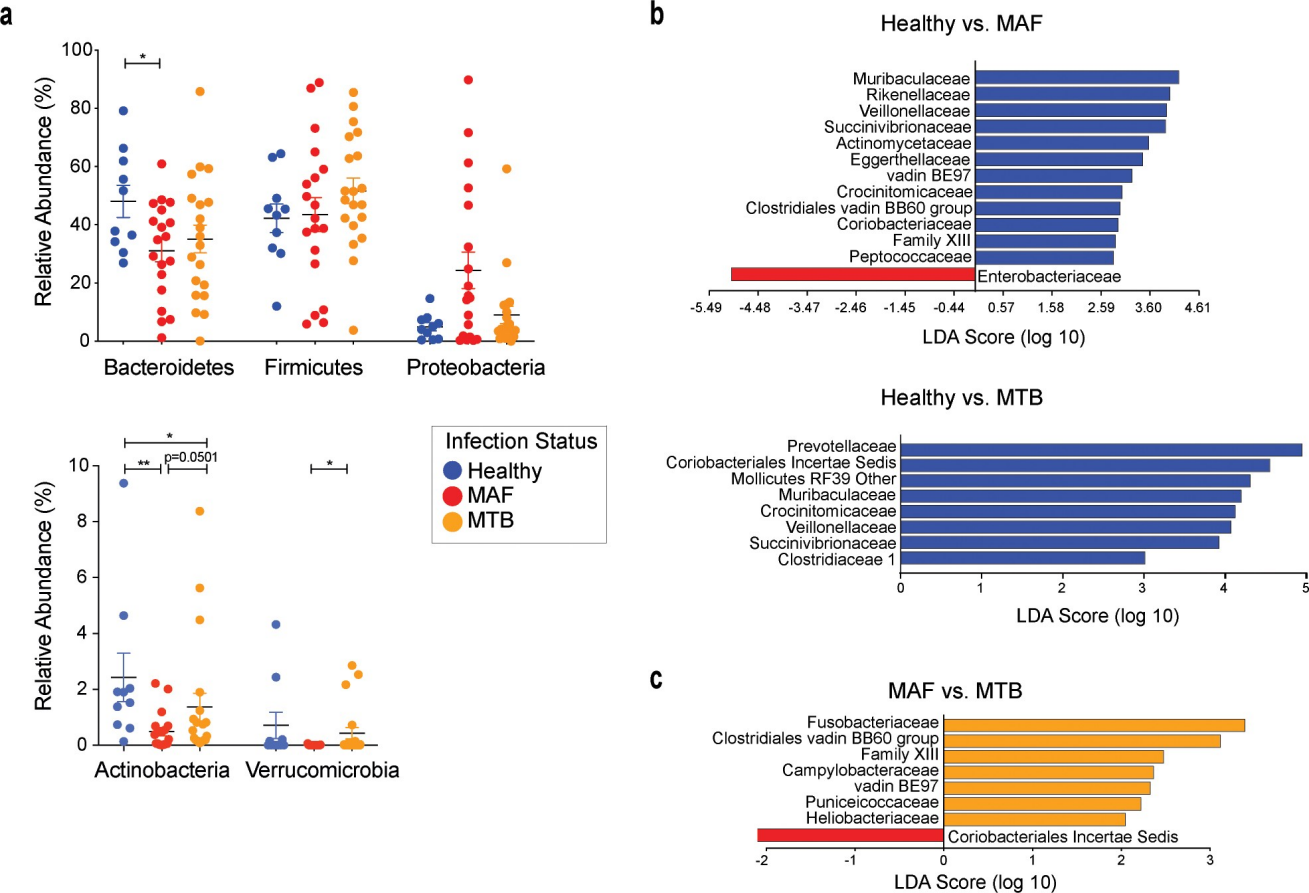
Alterations in the composition of the intestinal microbiome of MAF- and MTB-infected patients at the time of diagnosis. a. Relative abundances of the five most prominent phyla are compared between the three groups. Significant differences calculated using the non-parametric Mann-Whitney U test are indicated. *p < 0.05, **p < 0.01 (Significant differences identified using the parametric Student’s t-test are as follows: Bacteroidetes Healthy vs MAF p < 0.05, Proteobacteria Healthy vs MAF p < 0.05, MAF vs MTB p < 0.05, Actinobacteria Healthy vs MAF p < 0.01, Verrucomicrobia Healthy vs MAF p < 0.05). b-c. LEfSe analyses were performed to identify differentially abundant families in the two infection groups, MAF and MTB, compared to the healthy participants (b) and between the two infection groups (c). Taxa are filtered for p < 0.05 and linear discriminant analysis (LDA) score > 2.

In order to detect any specific compositional differences in the microbiome, we compared the bacterial taxa between the three groups. We observed a significant decrease in the relative abundance of phylum Bacteroidetes in the MAF-infected patients when compared to healthy controls, but not the MTB-infected patients. In the same analysis, no differences in the relative abundance of Firmicutes was observed. Phylum Proteobacteria was increased in MAF-infected patients when compared with either the MTB-infected patients or healthy controls when analyzed by the parametric Student t-test but not when compared utilizing the non-parametric Mann-Whitney U test (Fig 2A). While no significant differences were detected in phylum Firmicutes between the three groups, there was an overall 1.5 to 2-fold increase in the Firmicutes/Bacteroidetes ratio in the two TB-infected groups compared to the controls (S2A Fig). Among the phyla with lower abundance, levels of Actinobacteria were decreased in both MAF and MTB patients in comparison to healthy controls and were significantly different between the two infection groups. Additionally, phylum Verrucomicrobia was decreased in MAF-infected individuals in comparison to those with MTB infection (Fig 2A).

At the family level, a number of taxa were enriched in the healthy control group in comparison to both MAF- and MTB-infected patients with certain taxa such as Veillonellaceae, Succinivibrionaceae and Crocinitomicaceae increased in both comparisons (Figs 2B and S2B). We were also able to detect compositional differences in the MAF- versus MTB-infected patient comparison, with MTB-infected patients having relative increases in seven bacterial families (Figs 2C and S2B). Most importantly, the only taxon significantly increased in MAF-infected patients compared to healthy controls was Enterobacteriaceae of phylum Proteobacteria, a family consisting of a number of pathogenic bacteria. While Enterobacteriaceae levels in MTB patients were slightly elevated with reference to the healthy subjects and lower in comparison to the MAF group, these differences were not statistically significant (S2B Fig). Together, these observations revealed that MAF-infected patients display a decreased diversity and altered composition of their microbiota both in comparison to healthy controls as well as MTB-infected patients.

### Anti-tuberculosis treatment induces similar alterations in the composition of the microbiota of MAF and MTB infected patients

**Fig 3:**
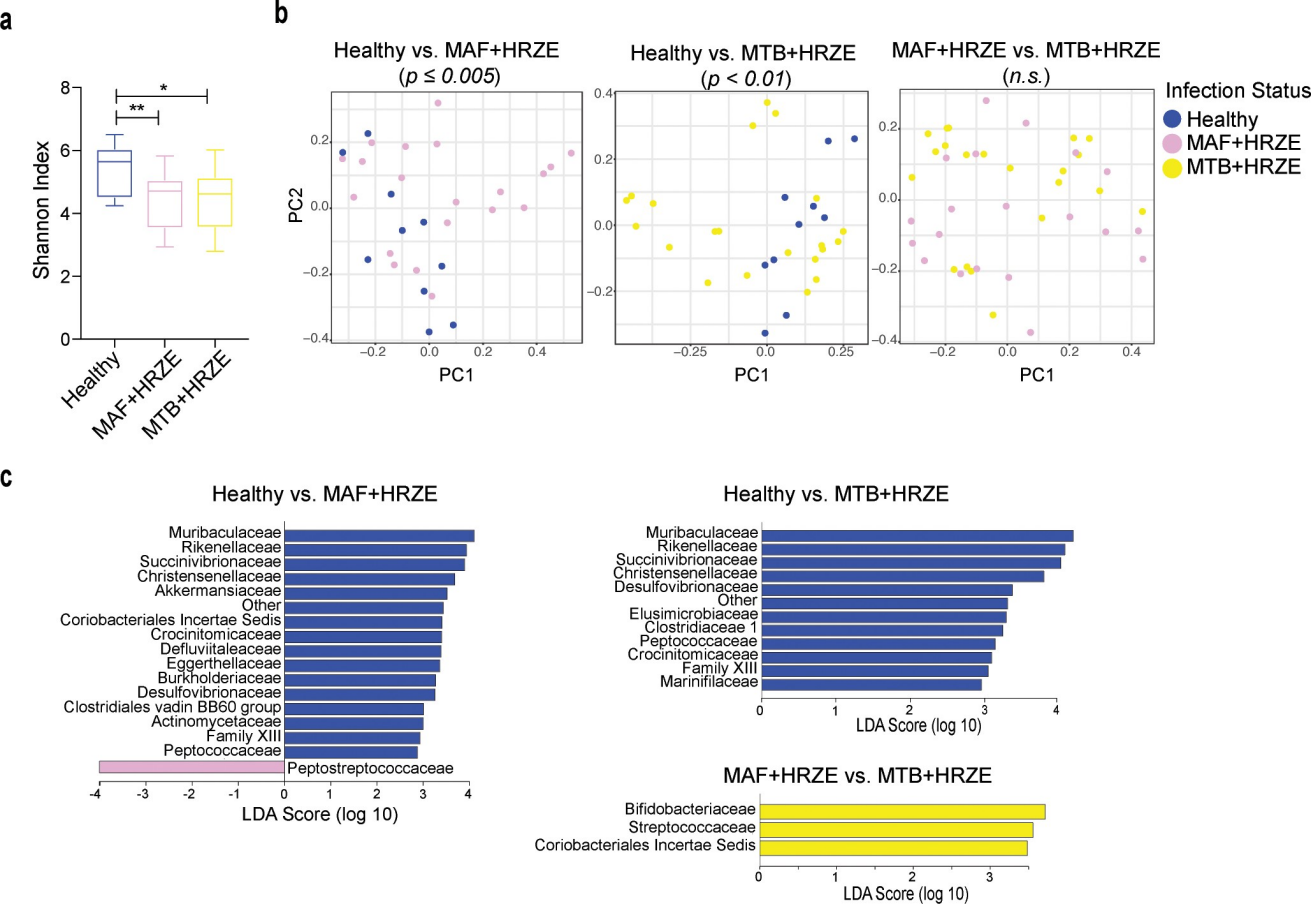
Perturbation in the intestinal microbiome composition due to HRZE treatment in both MAF and MTB infection. a. Alpha diversity was estimated following HRZE treatment in the two infection groups and compared to that of healthy controls and each other. Statistically significant differences are indicated. *p < 0.05 (Mann-Whitney U test). b. Pairwise beta-diversity clustering analysis were performed using the Bray-Curtis method and displayed on PC plots. Significance values are indicated if statistically significant (PERMANOVA with 999 permutations). c. Pairwise LEfSe comparisons were performed between the three groups to identify differentially abundant families. Taxa are filtered for p <0.05 and LDA score > 2.

While both MAF and MTB infections are treated with the same antibiotic regimen, a previous study has reported that MTB patients respond better to ATT which the authors proposed as resulting from a more optimal host immune response in comparison to MAF patients [6]. Therefore, it was of interest to investigate whether ATT differentially affects the intestinal microbiota in the two patient groups. In order to address this question, we examined the fecal microbiota two months following start of HRZE treatment in both MAF- and MTB-infected groups. Comparison of microbiota composition between the MAF- and MTB-infected as well as with the healthy controls were performed. In terms of alpha diversity, we observed that both TB-infected groups after ATT (MAF+HRZE and MTB+HRZE) displayed decreased microbiota diversity in comparison to untreated healthy controls (Fig 3A). However, the alpha diversities of the two TB-ATT groups did not differ from each other. Similarly, beta-diversity clustering analyses revealed that HRZE treatment caused a significant alteration in the microbiota community structure only in comparison to healthy controls (Fig 3B). Additionally, we observed that the bacterial community structure was similar between the two TB-infected and treated patient groups.

We next performed a three-way comparison among the groups to identify differences in microbiota composition. When comparing the microbiome of healthy controls to the MAF+HRZE or MTB+HRZE group, we found a number of the same taxa to be similarly enriched in the healthy controls (Fig 3C). These include Muribaculaceae, Rikenellaceae, Succinivibrionaceae, Christensenellaceae, Crocinitomicaceae and Peptococcaceae. Interestingly, some of these taxa were also found to be enriched in the healthy controls in comparison to TB-infected patients prior to ATT suggesting that these decreases in the TB disease groups were not due to treatment and likely occurred prior to or following the onset of active TB disease (Fig 2B, Fig 3C). Further, in line with the beta-diversity clustering analysis, we were able to identify only a few taxa that differed between the MAF+HRZE and MTB+HRZE groups with all three enriched in the MTB+HRZE group (Fig 3C).

**Fig 4:**
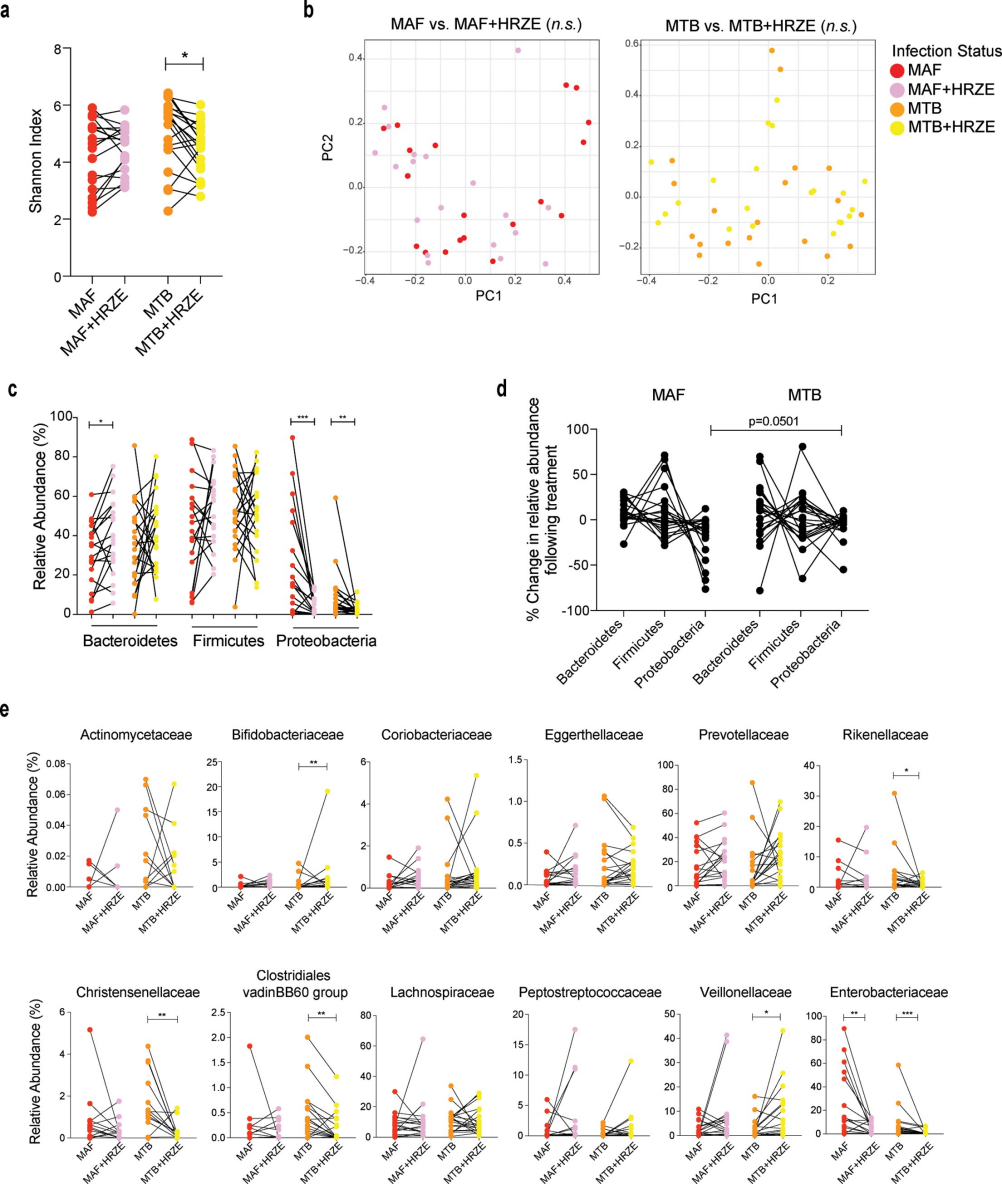
Longitudinal comparison of the microbiome in MAF- and MTB-infected patients before and after the initiation of anti-tuberculosis therapy. a. Paired comparisons of the Shannon alpha diversity index between pre- and post- two months of HRZE treatment microbiome of each patient in the MAF and MTB group were performed. Statistically significant difference is indicated. *p < 0.05 (paired Wilcoxon test). b. Beta-diversity between the pre- and post- treatment microbiome structure in the infection groups was estimated using the Bray-Curtis matrix. c. Relative levels of the three most abundant phyla are compared. d. Percent change in the relative composition of three phyla following two months of TB treatment are tracked for each patient. Significance estimates were calculated between the corresponding phyla of the two groups and statistically significant differences are indicated. e. Paired comparisons were performed to identify families that showed statistically significant change in relative abundance following antibiotic therapy. Select bacterial families are displayed. Statistically significant differences in c-e are indicated. *p < 0.05, ** p < 0.01, *** p< 0.001 (paired Wilcoxon test).

Utilizing a mouse model and longitudinal profiling, we previously observed that ATT results in largely similar changes in the microbiome among the different mice in the group [18]. However, the microbiota composition of inbred laboratory mice does not capture the heterogeneity observed in humans and thus it is unclear how each patient’s microbiome is affected due to treatment. The longitudinal nature of the present study enabled us to analyze each patient’s microbiome before and during ATT, a comparison which to the best of our knowledge has only been performed previously in a single study involving a smaller MTB-infected cohort [20]. We observed major inter-individual variability in antibiotic induced microbiome perturbations in both the MAF- and MTB-infected patient groups (Fig 4). A paired comparison of the alpha-diversity in each patient before and following two months of ATT revealed a significant decrease in diversity in the MTB- but not MAF-infected group (Fig 4A). Interestingly, we failed to observe a statistically significant difference in the beta-diversity clustering pattern of patients before and after treatment in either TB-infected group (Fig 4B). However, a phylum level comparison revealed a significant alteration in the relative proportions of Bacteroidetes and Proteobacteria following treatment in MAF-infected patients, which was primarily associated with an increase in Bacteroidetes and Firmicutes and a decrease in Proteobacteria (Fig 4C and 4D). At the family level, we observed ATT-induced changes in certain taxa in the MTB-infected group including a significant decrease in family Enterobacteriaceae (phylum Proteobacteria) (Fig 4E). Taken together, these findings indicate that ATT causes a heterogenous perturbation in the intestinal microbiome in both MAF- and MTB-infected patients with the most significant and consistent changes manifesting as decreases within the phylum Proteobacteria.

### Correlation between microbiome composition and blood transcriptome signature

One mechanism by which the microbiome is thought to influence host resistance is through its effects on metabolites that regulate immune responses [40]. To explore the existence of such a pathway in our MAF/MTB patients we looked for correlations between the microbiota composition and peripheral blood gene expression levels. Such transcriptomic profiles have been extensively studied in human TB as markers of disease staging and host immune response [30, 41]. To perform this preliminary analysis, we determined the blood transcriptome of the 10 healthy controls, and the first10 MAF- and 10 MTB-infected patients enrolled in the study by microarray (S1 Table). We then compared the signature obtained for each individual with that same patient’s microbiota composition prior to ATT. We began by analyzing the microarray data separately from the microbiome. In comparison to healthy controls, 1,970 genes were significantly upregulated, and 1,610 genes were downregulated in MAF-infected patients whereas 1,744 genes were upregulated, and 989 genes were downregulated in MTB-infected patients (S3A Fig). While a number of genes were uniquely up or down regulated in each group (S3A Fig), in agreement with a previously reported comparison of the transcriptomes of the MAF and MTB patients [42], we did not identify any significant differences in gene expression and the direction of modulation of genes was identical in both groups. In close agreement with previous studies on MTB [30, 43, 44], Gene Ontology (GO) term enrichment analysis and Ingenuity pathway analysis revealed a number of genes involved in metabolic and immune related pathways to be differentially regulated (S3B and S3C Fig) in both TB-infected groups in comparison to healthy controls.

**Fig 5:**
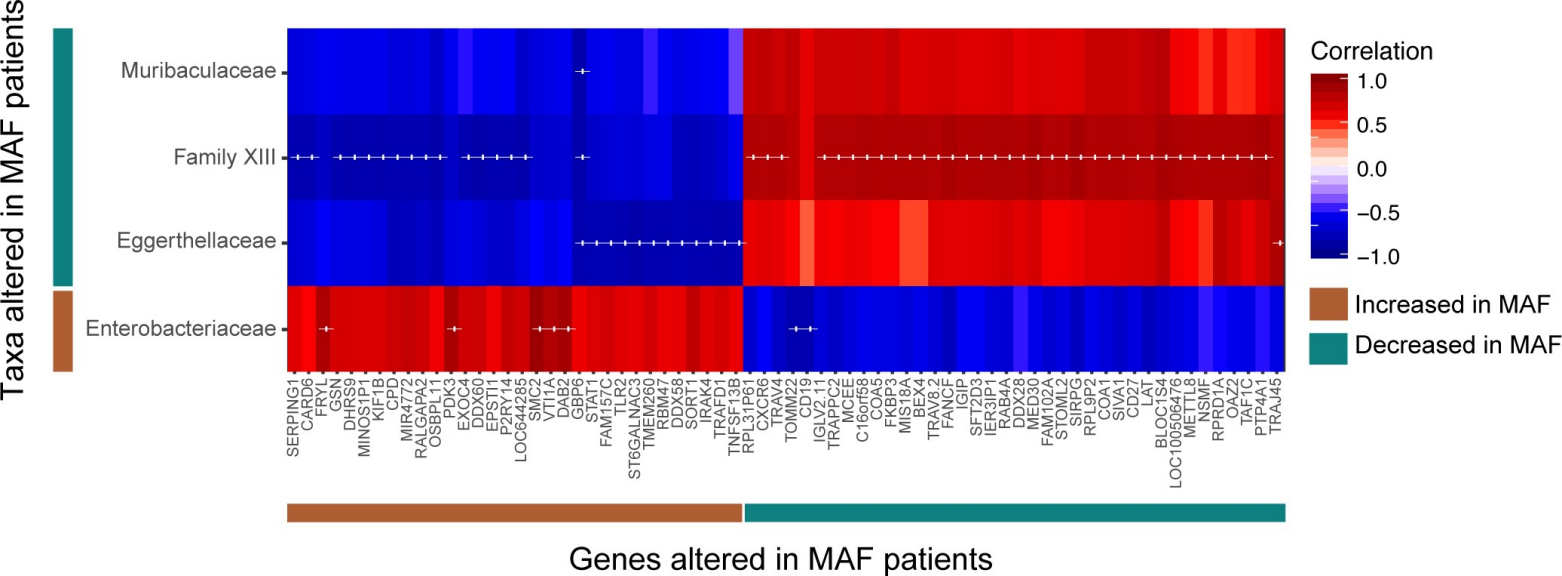
Correlation analysis between blood transcriptome profile and intestinal microbiome composition. Spearman correlation analyses was performed on a pool of 9 healthy and 8 MAF-infected subjects comparing the normalized expression of ~1600 immune-pathway related genes with the relative abundance of 13 bacterial families both parameters of which were altered during MAF infection in comparison to healthy individuals. These altered genes/taxa are identified as indicated in the key. Genes and taxa that had at least one significant correlation following Benjamini-Hochberg correction were selected. Unsupervised hierarchical clustering was performed on this correlation matrix and displayed as a heatmap. Red and blue indicate positive and negative correlations as indicated in the key. ‘+’ indicates a correlation with adjusted p-value ≦ 0.018 and was chosen instead of p < 0.05 for clearer visualization.

To search for associations between blood transcriptome profile and microbiome composition, we performed a paired correlation analysis using 26 participants (9 healthy controls, 8 MAF- and 9 MTB-infected patients) representing a subgroup of the entire study where both data sets were available. In the case of the MAF comparison, we used a pool of the 9 healthy and 8 MAF-infected patients and compared the relative abundance of the 13 taxa and ~1600 annotated immune-pathway related genes previously identified to be differentially abundant or expressed in MAF patients in comparison to healthy individuals. We identified a number of significant correlations between certain microbiota taxa and host blood transcripts (Fig 5). Specifically, Enterobacteriaceae that is enriched in MAF patients positively correlated with the expression levels of a set of genes upregulated during infection and negatively correlated with a gene set downregulated during the same infection (Fig 5). Conversely, taxa that are decreased in MAF patients in comparison to healthy individuals, Family XIII, Eggerthellaceae and Muribaculaceae, displayed the opposite trend. Of note, Family XIII that was also decreased in MAF when compared MTB patients displayed the highest number of significant correlations. In contrast, a similar correlation analysis performed on microbiota taxa and host genes altered during MTB infection, while exhibiting trends, did not reveal any significant correlations. Together, this exploratory analysis suggested that in a TB disease setting, relative increases in potential pathobionts such as those belonging to family Enterobacteriaceae positively correlate with the upregulation of immune-response related genes many of which are associated with inflammation.

## Discussion

TB caused by *M*. *africanum* is of great interest because of its primary restriction to one geographical region, West Africa. One hypothesis to explain this restriction is that West Africans are uniquely susceptible to infection with this *Mycobacterium* species. In this study, we asked whether there are differences in the intestinal microbiota of patients with MAF infection that could contribute to or reflect their disease susceptibility. We observed a significant decrease in microbiome diversity in MAF-infected patients compared to both MTB-infected patients and healthy controls. Perhaps more interestingly, bacteria belonging to phyla Actinobacteria and Verrucomicrobia were significantly decreased in the microbiota of patients with MAF versus MTB infection suggesting the existence of specific intestinal flora as markers distinguishing these two disease outcomes. Additionally, family Enterobacteriaceae of phylum Proteobacteria was enriched in MAF patients in comparison to healthy controls. In exploring this association, we observed a positive correlation between the relative abundance of Enterobacteriaceae and inflammatory gene expression by whole blood from infected patients in comparison to healthy controls. Together, these findings implicate the microbiome as a host-associated factor reflecting susceptibility to MAF versus MTB induced TB disease.

The present study also compared changes in the microbiome during ATT and found that the alterations caused by HRZE antibiotics were largely similar in the MAF- and MTB-infected patient groups. However, in agreement with previous reports involving treatment of MTB-infected patients alone, the microbiomes of treated TB patients in both groups were profoundly different from that of endemic healthy controls. Based on our previous studies utilizing mice treated with TB antibiotics, we hypothesize that these changes due to ATT occur irrespective of MAF or MTB infection status [18]. Importantly, the current study design allowed paired comparisons of the microbiota composition before and after two months of ATT. Although in most patients HRZE treatment resulted in a decrease in the relative abundance of Proteobacteria, the analysis revealed considerable inter-individual heterogeneity in the changes observed in the microbiota after ATT. While not directly addressed in the present study it would be interesting to analyze whether this heterogeneity correlates with the time required for successful mycobacterial clearance for each patient.

Both MAF- and MTB-infected patients displayed differences compared to healthy controls in the composition of their intestinal microbiota. In both TB patient groups, we observed a trend toward increased Firmicutes to Bacteroidetes ratio relative to healthy study participants. In agreement with a previously published Indian cohort study comparing the microbiomes of healthy household contacts with MTB patients, [20] we also observed lower levels of Prevotellaceae in MTB-infected patients and this decrease was absent in MAF-infected patients. Interestingly, other taxa found to be altered in the aforementioned Indian and a similar Chinese cohort study [19] were not identified as differentially enriched in healthy controls compared with MTB-infected patients in the present study. The non-overlapping nature of the taxa changes identified in the three regional cohorts (India, China and Mali) may stem from geographical, environmental and/or dietary induced differences in the populations studied. Taken together, these observations in human subjects support the conclusion of previous studies in murine models that MTB infection causes only minor alterations in the intestinal microbiome.

The key finding of the present study is the difference in the microbiota composition observed between MTB- and MAF-infected patients within the same endemic population. In general, MAF-infected patients displayed a more pronounced alteration of their intestinal flora in comparison to healthy controls than did MTB-infected patients. This was reflected in the microbiome diversity where MAF-infected patients displayed a significantly reduced Shannon index in comparison to healthy controls as well as to MTB-infected patients. In this regard, we noted significant decreases in the relative abundances of a number of taxa in the MAF versus the MTB or healthy groups. Nevertheless, as noted above the most prominent compositional difference was the significantly greater abundance of Enterobacteriaceae of phylum Proteobacteria in MAF-infected patients. Elevations in Proteobacteria associated with TB disease have previously been documented in one sputum study and in another report examining fecal microbiomes in a group of patients with recurring TB following ATT [19, 22]. In these studies, as well as in the data reported here, the observed overgrowth of Proteobacteria could have preceded the onset of or be the consequence of active TB disease.

Phylum Proteobacteria, while a component of the normal human gut microbiome, also includes a number of pathogenic bacteria. Indeed, numerous reports have associated increased Proteobacteria levels with metabolic and inflammatory disorders, certain cancers and intestinal dysbiosis [45–49]. For example, in murine spontaneous colitis models such as those utilizing Toll-like 5 receptor or interleukin-10 deficient mice, an overabundance of Proteobacteria was found to correlate with a heightened pro-inflammatory immune response [50, 51]. Similarly, in humans, patients with inflammatory bowel disease have been shown to display reduced intestinal microbial diversity accompanied by an overgrowth of Enterobacteriaceae of phylum Proteobacteria [48, 49, 52]. In this regard, in the present study we observed a positive correlative trend between the relative abundance of Enterobacteriaceae and inflammatory gene expression in host whole blood from MAF-infected patients. It is also of interest that levels of Proteobacteria were reduced following ATT. While this decrease may simply be the result of the direct action of the antibiotics on Proteobacteria, the observed reductions could also reflect the previously described effect of ATT in lowering host inflammatory gene expression [41] that might indirectly impact the levels of Proteobacteria.

The initial study reported here involved a single limited cohort of MAF- and MTB-infected patients in Mali, West Africa who were followed for only two months after the start of ATT. While the data revealed an association between the composition of intestinal microbiota and TB that was particularly prominent in the MAF-infected patients, these findings need to be validated in additional cohorts from different geographic regions with an extended characterization of clinical and demographic microbiome confounders. Similarly, the correlation observed between the inflammatory gene expression profile and microbiota composition needs to be confirmed in a larger cohort and supported by RNA-seq data. Nevertheless, the present study provides intriguing evidence suggesting a possible interaction (direct or indirect) of the microbiota and TB disease. In this regard, a recent study utilizing a rhesus macaque model of MTB infection demonstrated that the composition of the base-line, pre-infection microbiota correlates with TB disease severity [53]. Studies with a similar design where patients are sampled prior to the development of either MAF and MTB disease would be needed to formally establish this association between the microbiota and these two distinct mycobacterial infections.

## Conclusion

Previous studies in mouse models employing broad-spectrum antibiotic or bacterial induced dysbioses have indicated a role for the intestinal microbiota in influencing susceptibility to experimental MTB infection. However, the importance of the microbiome in human TB has been less clear. By comparing the intestinal microbiomes of patients infected with either MTB or the co-endemic hypovirulent MAF species, we have obtained new evidence supporting an association of the microbiota with susceptibility to TB and, in the case of MAF, implicating increased abundance of Enterobacteriaceae as a prominent feature of this relationship. Whether the observed association reflects a direct influence of the microbiota or is merely an indirect biomarker of other host differences is presently unclear. Further studies that both confirm this association and examine whether the microbiota signature found in MAF-infected patients is predictive of or the consequence of disease onset will be important in determining the physiological and potential clinical significance of these initial observations.

## Supporting information

S1 Fig Beta-diversity comparisons of characteristics of the study cohort in healthy, MAF and MTB groups. Pairwise beta-diversity estimates were calculated between the two groups indicated using the Bray-Curtis dissimilarity index and presented here as a principal component (PC) plot. Each circle denotes a single patient and is color-coded by the demographic or clinical parameter compared and is indicated in the key for each comparison. Statistical significance was calculated using PERMANOVA with 999 permutations and is indicated for each comparison (*n*.*s*.–not significant).
(TIF)

S2 Fig Comparisons of relative abundances of specific intestinal microbial taxa in healthy, MAF and MTB groups. a. Firmicutes/Bacteroidetes ratio for each patient in the study groups were calculated using the relative abundances of the two phyla and plotted. One patient in the MTB group was dropped in this figure for display purposes as this participant’s ratio was 441 and clearly off scale in comparison to the other subjects. Differences in ratio between groups were not statistically significant. b. Relative abundances of the families shown in Fig 2B and 2C are displayed. Significant differences indicated here were calculated using the Mann-Whitney U test. *p < 0.05, **p < 0.01, ***p < 0.001.
(TIF)

S3 Fig Differential gene-expression in MAF- and MTB-infected patients in comparison to healthy participants. a. Venn-diagram depicting the number of genes that were significantly up- and down-regulated in the MAF and MTB groups in comparison to healthy individuals. Genes that displayed a fold change of > 2 and Benjamini-Hochberg corrected p-value of < 0.01 were considered significant. b. Ingenuity pathway analyses were performed on the differentially expressed genes and the top 15 canonical pathways that were differentially modulated are displayed. The direction of change in shown as indicated in the key. c. Unsupervised hierarchical clustering was performed on 50 immune-pathway related genes with the most fold-change in either direction (up or down) and visualized as a heat map. The participants color-coded by group are indicated on top along the x-axis and genes are clustered along the y-axis. Green and orange indicate increase or decrease in gene expression respectively.
(TIF)

S1 Table Sociodemographic characteristics of participants that underwent the transcriptome analysis. (DOCX)
