# Supplementary figures and images for: Patients infected with Mycobacterium africanum versus Mycobacterium tuberculosis possess distinct intestinal microbiota
Source: PLoS Negl Trop Dis. 2020 May 13;14(5):e0008230. doi: 10.1371/journal.pntd.0008230 (PMC7219701; doi:10.1371/journal.pntd.0008230)

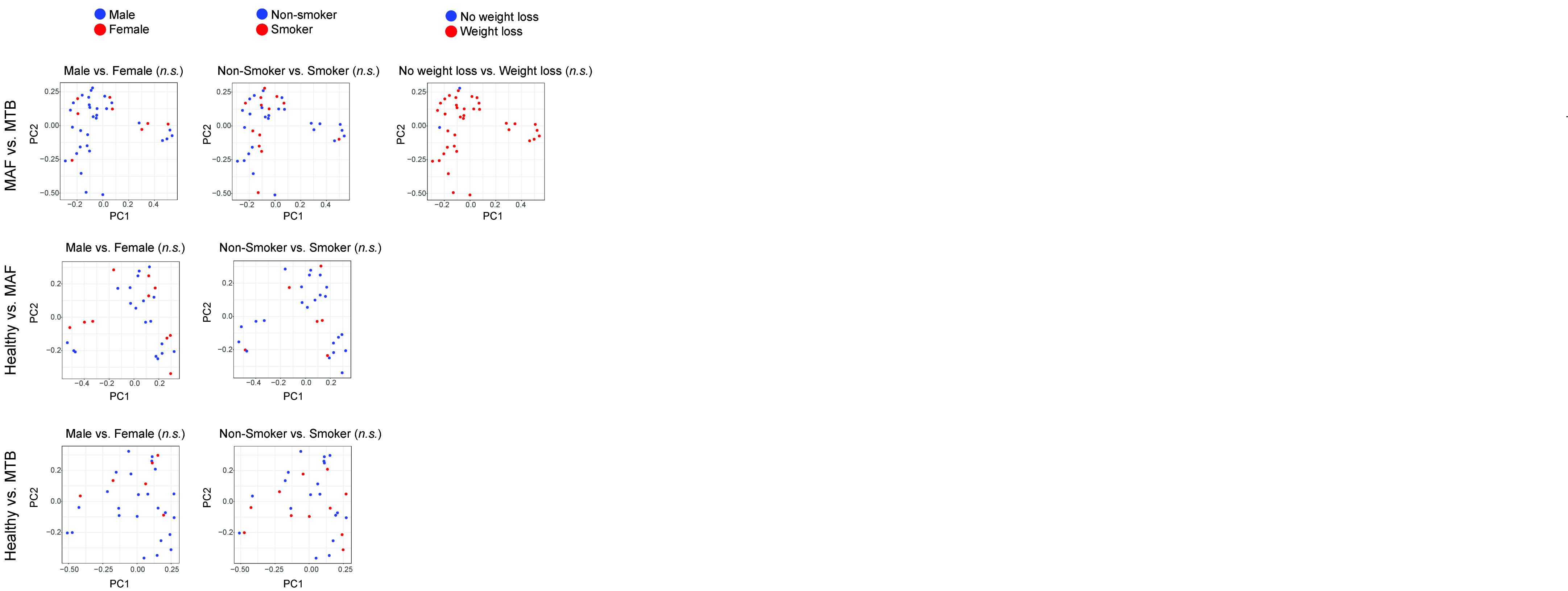

Supplement: S1 Fig — Pairwise beta-diversity estimates were calculated between the two groups indicated using the Bray-Curtis dissimilarity index and presented here as a principal component (PC) plot. Each circle denotes a single patient and is color-coded by the demographic or clinical parameter compared and is indicated in the key for each comparison. Statistical significance was calculated using PERMANOVA with 999 permutations and is indicated for each comparison (n.s.–not significant). (TIF) [file pntd.0008230.s001.tif]

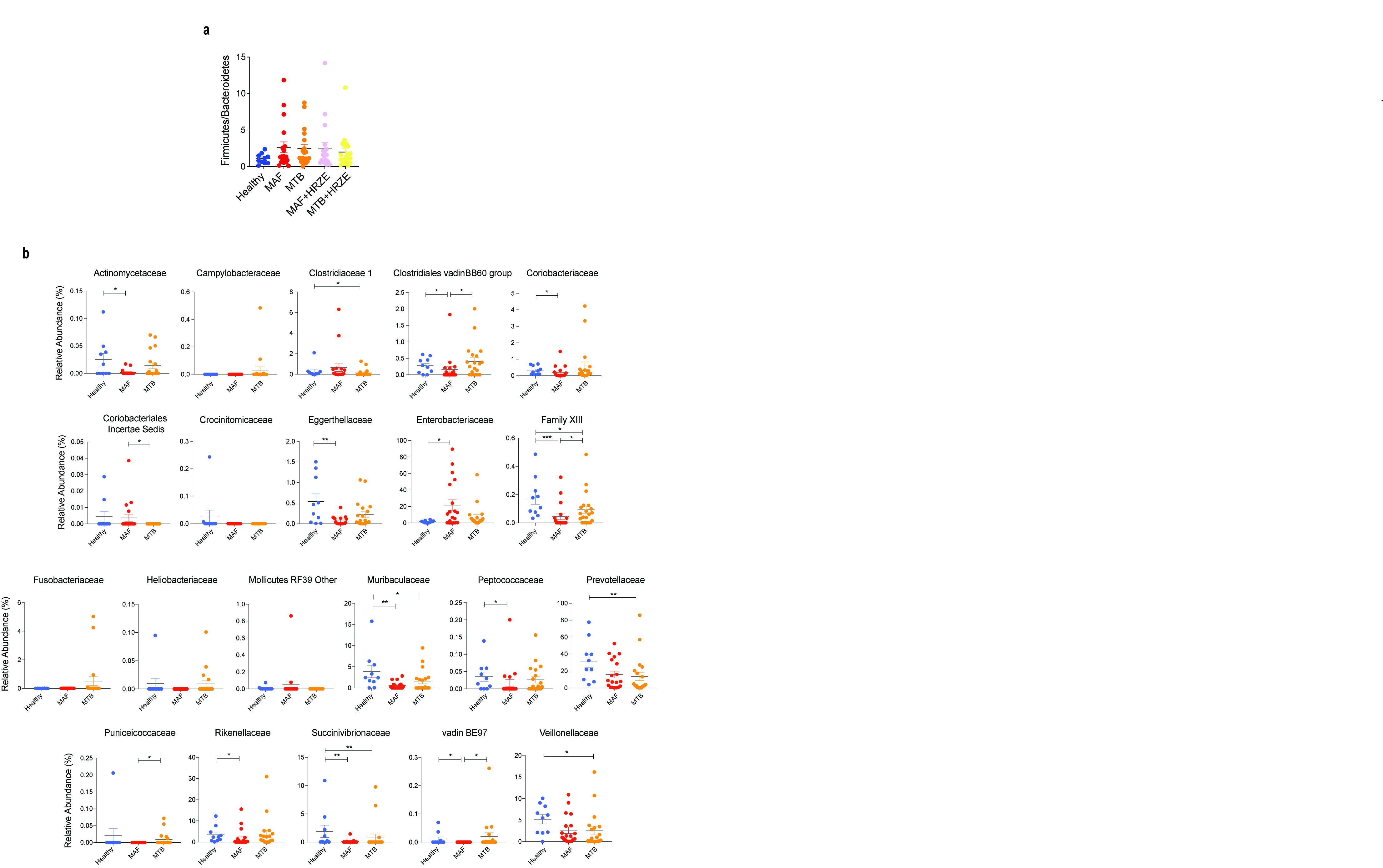

Supplement: S2 Fig — a. Firmicutes/Bacteroidetes ratio for each patient in the study groups were calculated using the relative abundances of the two phyla and plotted. One patient in the MTB group was dropped in this figure for display purposes as this participant’s ratio was 441 and clearly off scale in comparison to the other subjects. Differences in ratio between groups were not statistically significant. b. Relative abundances of the families shown in Fig 2B and 2C are displayed. Significant differences indicated here were calculated using the Mann-Whitney U test. *p < 0.05, **p < 0.01, ***p < 0.001. (TIF) [file pntd.0008230.s002.tif]

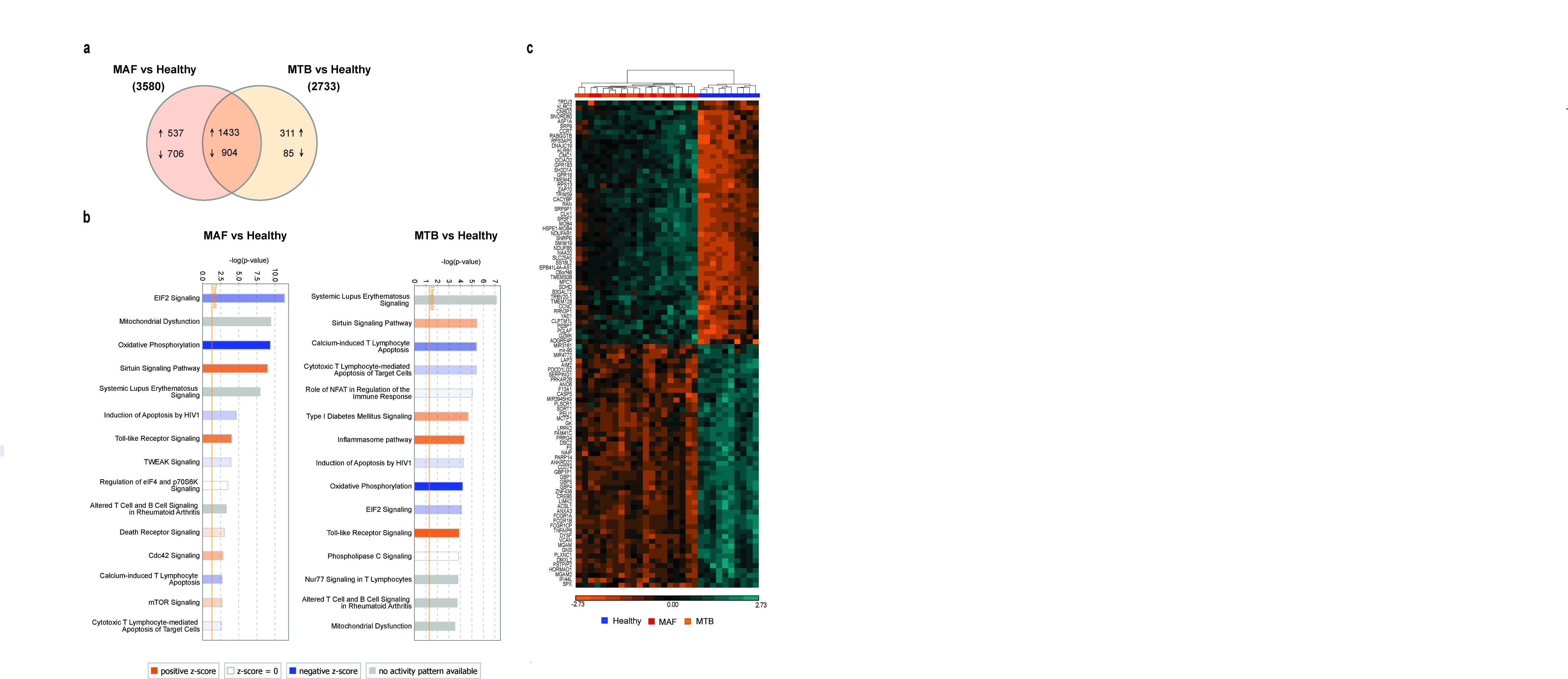

Supplement: S3 Fig — a. Venn-diagram depicting the number of genes that were significantly up- and down-regulated in the MAF and MTB groups in comparison to healthy individuals. Genes that displayed a fold change of > 2 and Benjamini-Hochberg corrected p-value of < 0.01 were considered significant. b. Ingenuity pathway analyses were performed on the differentially expressed genes and the top 15 canonical pathways that were differentially modulated are displayed. The direction of change in shown as indicated in the key. c. Unsupervised hierarchical clustering was performed on 50 immune-pathway related genes with the most fold-change in either direction (up or down) and visualized as a heat map. The participants color-coded by group are indicated on top along the x-axis and genes are clustered along the y-axis. Green and orange indicate increase or decrease in gene expression respectively. (TIF) [file pntd.0008230.s003.tif]
