# Supplementary material for: Patients infected with Mycobacterium africanum versus Mycobacterium tuberculosis possess distinct intestinal microbiota
Source: PLoS Negl Trop Dis. 2020 May 13;14(5):e0008230. doi: 10.1371/journal.pntd.0008230 (PMC7219701; doi:10.1371/journal.pntd.0008230)
Supplement: S1 Table — (DOCX) [file pntd.0008230.s004.docx]

**S1 Table. Sociodemographic Characteristics of Participants that underwent the Transcriptome analysis.**

| **Parameters** |  | ***M. tuberculosis***  **N=9**  **n (%)** | ***M. africanum***  **N=8**  **n (%)** | **Controls**  **N=10**  **n (%)** | **p-value^$^** |
| --- | --- | --- | --- | --- | --- |
| **Gender - Male** |  | 7 (77.78) | 4 (50.00) | 6 (60.00) | 0.768 |
| **Age** | [18-30] | 6 (66.67) | 2 (25.00) | 8 (80.00) | -* |
|  | [30-45] | 3 (33.33) | 4 (50.00) | 1 (10.00) | -* |
|  | [45-60] | 0 (0) | 1 (12.50) | 1 (10.00) | -* |
|  | [60-75] | 0 (0) | 1 (12.50) | 0 (0) | -* |
| **Smoking (current and past)** | Yes | 2 (22.22) | 2 (25.00) | n/a | -* |
| **Inner-city or sub-urban** | Yes | 8 (88.89) | 7 (87.50) | 9 (90) | 0.935 |
| **Household contacts** | Yes | 1 (11.11) | 3 (37.50) | n/a | -* |
| **Sputum smear at M0^#^** | Many AFB | 9 (100.00) | 7 (87.50) | n/a | 0.298 |
| **Sputum smear at M2^#^** | Moderate AFB | 4 (44.44) | 2 (25.00) | n/a | -* |
|  |  |  |  |  |  |
| **Sputum culture at M0^#^** | Positive | 9 (100.00) | 8 (100.00) | n/a | - |
| **Sputum culture at M2^#^** | Positive | 0 (0) | 1 (12.50) | n/a | -* |
| **Chest X-rays** | Bilateral infiltrate | 0 (0) | 3 (37.50) | n/a | -* |
|  | Cavitary lesions | 3 (33.33) | 1 (12.50) | n/a | -* |
|  | Miliary pattern | 0 (0) | 0 (0) | n/a | -* |
|  | Unilateral infiltrate | 1 (11.11) | 3 (37.50) | n/a | -* |

^$^p-value was calculated using Chi-square test

*p-value not calculated when n was less than 5

^#^M0- At the time of diagnosis before start of ATT; M2- Two months after start of ATT
